# Dynamic Complexity of Positive and Negative Affect in NSSI – A Daily Diary Study

**DOI:** 10.32872/cpe.14527

**Published:** 2025-05-28

**Authors:** Michaela Bruckbauer-Schwed, Tim Kaiser, Marc Keglevic, Anton-Rupert Laireiter

**Affiliations:** 1 Department of Psychiatry, Kardinal Schwarzenberg Klinikum, Schwarzach, Austria; 2 Department of Psychology, University of Salzburg, Salzburg, Austria; 3 Department of Methods and Evaluation / Quality Assurance, Free University Berlin, Berlin, Germany; Philipps-University of Marburg, Marburg, Germany

**Keywords:** non-suicidal self-injury (NSSI), affect instability, positive affect, dynamic complexity, ecological momentary assessment

## Abstract

**Background:**

Non-suicidal self-injury (NSSI) is a major health problem. Functionally, it is related to affect instability and increased affective intensity. The role of negative emotions has already been extensively explored, only few studies have focused on positive emotions. The concept of dynamic complexity (DC) is particularly well suited to differentially analyze the dynamics of affect collected by ecological momentary assessment (EMA). This study examines DC of positive and negative emotions in individuals with and without NSSI history in an EMA setting.

**Method:**

Participants from a clinical NSSI group (*n* = 28) and a comparable clinical non-NSSI control group (*n* = 33) completed the Positive and Negative Affect Schedule (PANAS) once a day between six to 37 days (*M* = 15.60, *SD* = 5.80). DC was calculated for the assessed time-series of daily affect. Additionally, we fitted a linear mixed model to predict positive and negative dynamic complexity with length of stay and group.

**Results:**

Compared to controls, individuals with a history of NSSI showed significantly more positive affect and had significantly higher DC in affect in general. No significant difference for negative affect was found.

**Conclusion:**

Our results suggest that it is important to assess dynamic emotional patterns and to analyze in detail the role of positive and negative affect in individuals with NSSI in order to better understand the complex interplay between the different emotional states and to be able to use it for diagnostic purposes and clinical interventions.

## Highlights

- The NSSI group showed a higher DC in positive and negative emotions than clinical controls.
- The NSSI group showed higher mean levels of positive emotions than clinical controls.
- Within-person variation in DC explains more variance than survey period or group differences.

Non-suicidal self-injury (NSSI) is a serious psychiatric phenomenon characterized by the direct, intentional destruction of bodily tissues without the intent to die (Nock & Favazza, 2009). It is a growing public health concern worldwide due to its high prevalence rates (Muehlenkamp et al., 2012) and increased emergency department utilization (Olfson et al., 2005). In inpatient samples, 21% of adults report a history of NSSI (Briere & Gil, 1998).

It is now well established from various studies that emotion regulation (Gratz & Roemer, 2008) is one of the most commonly endorsed functions of NSSI. Emotion regulation refers to the methods individuals use to manage their emotions, including determining which emotions they experience, when they occur, and how they are felt and expressed (Gross, 1998). Klonsky (2009) found that 85% of participants engaged in NSSI to alleviate negative emotions, such as sadness, frustration, and pain and reported feeling calm and relieved afterwards. Claes et al. (2010) also found similar results, where participants reported a decrease in negative high arousal emotions, such as anxiety and depression, and an increase in positive-low arousal emotions, such as relief.

In addition to emotion regulation, affect instability also plays an important role in NSSI (Kerr & Muehlenkamp, 2010). Affect instability is defined as rapid and intense mood swings with difficulties in controlling these swings and their outcomes (Marwaha et al., 2014). Many studies (e.g., Peters et al., 2016; Santangelo et al., 2017) reported an increased affect instability in individuals with NSSI compared to controls. Furthermore, affect instability predicts the onset and continuation of non-suicidal self-injury (Peters et al., 2016).

Another aspect of impaired emotion regulation is variability of positive and negative affect. Affect variability pertains to the extent or magnitude of an individual's affective states over a period of time. If someone exhibits higher levels of affective variability, he/she experiences emotions that are more intense and deviate more significantly from his/her typical affective state (Houben et al., 2015). Ong and Steptoe (2020) reported that greater variability in positive affect increased mortality risk in older adults. Conversely, in younger adults, instability in positive affect was related to better mental health (Spindler et al., 2016). Victor et al. (2021) demonstrated that variability in negative affect was associated with NSSI, suicidal ideation, and suicidal behavior assessed concurrently as well as prospectively.

There is also limited knowledge about changes in emotion dysregulation over the course of inpatient hospitalization. Few studies have examined this issue in adult inpatients. For example, Fowler et al. (2016) found significant improvements in experiential avoidance and emotion dysregulation with large effect sizes following intensive inpatient psychiatric treatment over a period of six to eight weeks.

A common idea in various theoretical models is that individuals engage in NSSI in response to intense emotional distress aiming to regulate or alleviate these distressing states by modifying, reducing, or diverting attention from them in some way (e.g., Nock & Prinstein, 2004; Selby & Joiner, 2009). An approach to explain the development and maintenance of self-injury has been proposed by Nock and Prinstein (2004) with their four-factor model based on learning theory. This functional approach describes four reinforcement processes: automatic negative reinforcement when NSSI is performed to alleviate negative states, automatic positive reinforcement when NSSI is followed by an increase in positive affect, social negative reinforcement when NSSI is performed to reduce or stop interpersonal demands, and social positive reinforcement when NSSI is performed to seek attention or receive help.

Much of the research to date has focused on the negative reinforcements associated with NSSI. Only a few studies have found an association between positive affect and NSSI. Some studies have found an increase in positive affect following NSSI (Jenkins & Schmitz, 2012; Kranzler et al., 2018) However, these self-report and ecological momentary assessment data on positive affect need to be replicated. In addition, motives for engaging in NSSI behaviors seem to include attempts to reduce negative affect and increase positive affect (Claes et al., 2010; Selby et al., 2014). Regarding the four-factor model, Yen et al. (2016) reported that adolescents in psychiatric inpatient settings who endorsed automatic positive reinforcement as a motive for NSSI were more likely to continue with NSSI. Turner et al. (2012) reported that 92% of an adult sample with a history of NSSI engaged in self-injury as a means of generating positive emotions. Other studies have found no support for an increase in positive affect after engaging in NSSI (Armey et al., 2011) and no group differences in positive emotional reactivity in individuals with NSSI compared to controls (Mettler et al., 2021). Jenkins and Schmitz (2012) showed that positive, but not negative affect, was significantly associated with a higher frequency of lifetime NSSI acts in college students.

## Dynamic Complexity (DC)

A growing body of literature investigated the relationship between NSSI and affect with correlational and ANOVA-based analyses (e.g., Victor & Klonsky, 2014). However, no previous study was grounded in a nonlinear-dynamic systems framework where it is important to consider dynamic characteristics (such as fluctuations or critical slowing down) of affect and its relation to NSSI.

According to the synergetic approach to psychology, human change processes are characterized by non-linearity and non-stationarity of their dynamics (Schiepek & Strunk, 2010). When the relationship between input and output of a dynamic system is not directly proportional, the system is considered nonlinear (Stam, 2005). It is typical for nonlinear dynamic systems that, depending on the state of the system, even high levels of negative affect (input) do not lead to non-suicidal self-injury (output). In contrast, when the system is in an unstable state, even minor increases of negative affect can trigger self-injurious behavior. Nonstationarity, on the other hand, denotes changes in descriptive statistics, including (moving) averages and variance, over time. Nonstationarity is consistent with discontinuous phase transitions caused by the nonlinear mechanisms inherent in complex systems. Phase transitions are defined as a change from one pattern to another pattern, caused by internal and external conditions. Almost invariably, phase transitions are preceded and facilitated with critical instabilities (Schiepek & Strunk, 2010).

**Figure 1:**
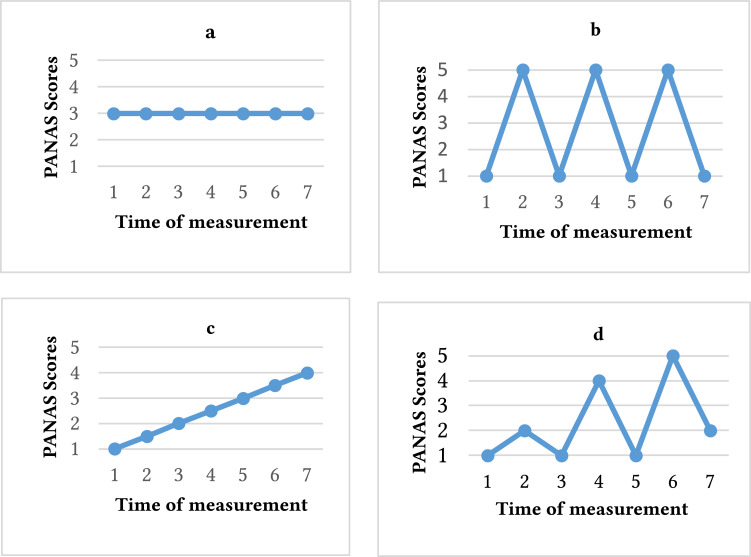
Diagrammatic Illustration of Various Modes of Distribution, Fluctuation, and Dynamic Complexity in One Individual *Note.* Distribution describes how the time series data is distributed across the scale range, fluctuation: frequency and amplitude of changes in time series, dynamic complexity: multiplicative product of a fluctuation measure and a distribution measure, *b* illustrates the highest fluctuation, while *a* illustrates the lowest one. *c* and *d* illustrate the highest distribution, *a* illustrates the lowest distribution. *b* and *d* show high dynamic complexity, whereas *a* shows low dynamic complexity.

Critical instabilities preceding phase transitions can be assessed with the dynamic complexity (DC), which was developed for short time series typical in psychological research (Schiepek et al., 2014). DC is the product of two parameters, fluctuation (F) and distribution (D). F quantifies the intensity of change with respect to frequency and amplitude of a time-series (Figure 1). F is maximal when the variable in the time-series jumps between the minimum and maximum value as often as possible. D quantifies the scattering of the time-series within the range of possible values. D maximizes when values are irregular and chaotically distributed (for a detailed description and formula, see Schiepek & Strunk, 2010). Consequently, high DC indicates frequent, large, and irregular fluctuations (Olthof et al., 2020).

F and D and their product DC indicate critical instabilities in self-organizing processes and non-stationary phenomena in time series (Schiepek & Strunk, 2010). During periods of fluctuation, the system is destabilized and oscillates between old and new patterns until the system stabilizes in a new state (attractor) and variability decreases (Kelso, 1995; Thelen & Smith, 1994). Typically, DC is computed by looking at a window of data consisting of seven measurement points that are shifted from one day to the next throughout the time series. Applying this procedure produces a time series that includes DC values for each item (Schiepek & Strunk, 2010). A manifestation of nonlinear dynamics and phase transitions is the rise and fall in affect intensity over time. Individuals engaging in NSSI typically show high affective variability (Victor et al., 2021), which reflects the overall amplitude of affective changes. Affective changes (from positive to negative affective states) can be considered as order transitions. The typical high affect fluctuations in individuals engaging in NSSI could be recognized by the presence of more critical instabilities in their affect data. Therefore, we hypothesize that an order transition phenomenon like affective change is accompanied by phases of critical instabilities (fluctuations in affect) of the time series resulting in an increase of dynamic complexity in individuals with a history of NSSI compared to controls.

To summarize, research has proposed that NSSI is associated with intense negative affect and affective instability. Although the evidence has highlighted the role of emotion regulation in NSSI through negative reinforcement of aversive affect (e.g., Nock & Prinstein, 2004), some research has examined positively reinforcing effects (e.g., Jenkins & Schmitz, 2012).

## Present Study

Based on the literature described above, the present study is the first to apply the concept of DC to the description and analysis of NSSI. We are particularly interested in the following three hypotheses: First, we expect individuals engaging in NSSI to show a higher DC than controls without a history of NSSI. The resulting high DC in the NSSI group should be represented by frequent, large, and irregular fluctuations in time series. Second, we aim to examine mean levels of positive and negative affect in the NSSI group compared to controls. Previous research mostly focused on the role of negative affect and negative reinforcement in NSSI. Therefore, one could assume that participants with NSSI also show higher negative affect. However, we also wanted to investigate whether positive affect might be present as well. So, investigating DC in affective states that differ between individuals with and without a history of NSSI may be an opportunity to identify potential maintaining or risk factors of NSSI. Third, we investigate whether the average values of dynamic complexity change over time. We assume that the dynamic complexity should decrease during the course of the hospital stay, as therapeutic and pharmacological interventions should lead to the stabilization of the patients.

## Method

### Daily Assessments

Ecological momentary assessment (EMA) enables an assessment of behavior and psychological processes in everyday life. EMA reduces the influence of recall biases, enhances research findings' ecological validity, and tracks changes in intra-individual psychological processes (Stone & Shiffman, 1994). Another advantage of EMA is the distinction between state affect and trait affect.

### Participants

Inclusion criteria were a) suffering from a mental disorder; b) aged between 18 and 65 years; c) having adequate language skills in German to understand and follow instructions and give informed consent; d) non-suicidal self-injury in the last year for the NSSI-group and no such behavior in the control-group. Exclusion criteria were a) acute episodes of psychosis, psychotic or manic episodes, rapid cycling, and acute substance use; b) missing cognitive skills to complete assessment.

61 subjects selected according to the procedure described below met the required criteria, 33 (54.1%) of them did not show NSSI and constitute the control group (22 women, 11 men) with a mean age of 35.0 (*SD* = 10.73) years. In this group, 18 (54.5%) met the diagnostic criteria (ICD-10) of a depressive disorder, ten (30.3%) of an anxiety disorder, two (6.1%) of an obsessive-compulsive disorder, two (6.1%) of a personality disorder (avoidant and dependent personality disorder), and one (3.0%) of insomnia. The NSSI group consisted of 28 (45.9%) participants (26 females, two males) with a mean age of 26.69 (*SD* = 8.73) years. Of these, 12 (42.9%) met ICD-10 criteria for a depressive disorder, eight (28.6%) for a post-traumatic stress disorder, five (17.9%) for a primary diagnosis of borderline personality disorder, and three (10.7%) for an eating disorder. In addition, eight (28.6%) were diagnosed with comorbid borderline personality disorder. The diagnoses were initially assigned by the treating psychiatrist based on the clinical picture and confirmed in a second step by the researcher using the ICD-10 research criteria.

### Procedure

Subjects were recruited from an Austrian adult inpatient psychiatric unit (“Kardinal Schwarzenberg Klinik”) located in the southern area of the federal state of Salzburg. Patients meeting inclusion criteria were invited to participate in the study and were informed about the studies’ procedure in detail and written informed consent was obtained. Additionally, the phone number was recorded for being able to contact subjects during the study. Prior to commencement, the study procedure was approved by the Ethics Committee of the Province of Salzburg (EC number: 415-E). Only the third hypothesis was preregistered (see Bruckbauer-Schwed et al., 2023S), as the data for the first and second hypotheses had already been analyzed at the time of registration. Data collection started with a general questionnaire asking for age, gender, professional and family status and NSSI. With respect to that, subjects were asked if ever in their lives they had performed any NSSI-behavior; if yes, they were additionally asked about its frequency over their lifetime. Positive and negative affect was recorded by using the Positive and Negative Affective Schedule (PANAS) in an EMA-design over a period of at least six days. Participants received no compensation for their participation.

EMA was initiated by an invitation by a SMS-message. The questionnaire could be retrieved and completed in SoSci Survey (https://www.soscisurvey.de) on the subjects’ mobile phones. Due to technical problems, data acquisition had to be changed to the mobile PIEL App (Jessup et al., 2012). With a wake-up function subjects were prompted to answer their questionnaires at 6 p.m. daily taking approximately five minutes to be completed. For assistance participants could contact the researcher at any time during the study period. It was also possible to complete the questionnaire in a paper-pencil-format. Two participants choose this option for some of their entries.

### Positive and Negative Affective Schedule (PANAS)

The Positive and Negative Affective Schedule (PANAS; Watson et al., 1988; German: Krohne et al., 1996) consists of two 10-item scales, one for positive and one for negative affect. Participants rated, how they generally felt during each day with respect to each emotion on a 5-point Likert-like intensity scale (1 = *very slightly/not at all*, 5 = *extremely*). The internal consistency of the PANAS is high, with Cronbach alpha’s of 0.90 for positive and 0.87 for negative affect (Watson et al., 1988).

### Statistical Analyses

Dynamic complexity (DC) (Schiepek & Strunk, 2010) was computed for the PANAS´s positive and negative affect subscales. DC is a measure for identifying critical instabilities and fluctuations in short, coarse-grained, time series like those obtained in typical diary studies. It is calculated from two parameters: fluctuation intensity and distribution (see section on DC above). The fluctuation measure is highest when the time series fluctuates widely between its minimum and maximum, while the distribution measure is larger when it takes many different values. Both parameters are then multiplied by each other, resulting in a total dynamic complexity measure. Both parameters were calculated in moving windows. Like other studies using this method (e.g., Schiepek et al., 2014), we chose a moving window size of seven days. To identify local complexity peaks, we calculated the difference between the average and maximum complexity of each PANAS item. Peak complexity values for individual positive and negative affect items were averaged to obtain scale-level peak complexity values. We then used *t*-tests to compare the peak complexity values for the positive and negative affect scales of patients with NSSI to those of controls. Finally, to test whether average dynamic complexity values change over time, we fitted linear mixed models to predict dynamic complexity of the positive and negative affect scales from number of days since admission and group (NSSI vs. controls). Models included random intercepts for each participant and an interaction effect of the group by time. The linear mixed models were included to determine whether there were temporal trends in the development of dynamic complexity. Missing values were imputed using Kalman filter imputation.

## Results

The assessment period for the NSSI group was between 8 and 37 days (*Mdn* = 16.5, *M* = 17.21, *SD* = 5.56) and between 6 and 32 days for the control group (*Mdn* = 14.0, *M* = 14.24, *SD* = 5.73). The frequency of NSSI acts during their lifetime was classified in intervals. Eleven participants indicated more than 60 NSSI acts, three between 31 and 60, seven between 30 and 16, three between six and 15, and four between two and five acts. In the NSSI group, a median number of 2.5 assessments (interquartile range: 5.25) were missing and had to be imputed. In the control group, a median of 3 assessments (interquartile range: 4) were missing.

**Figure 2:**
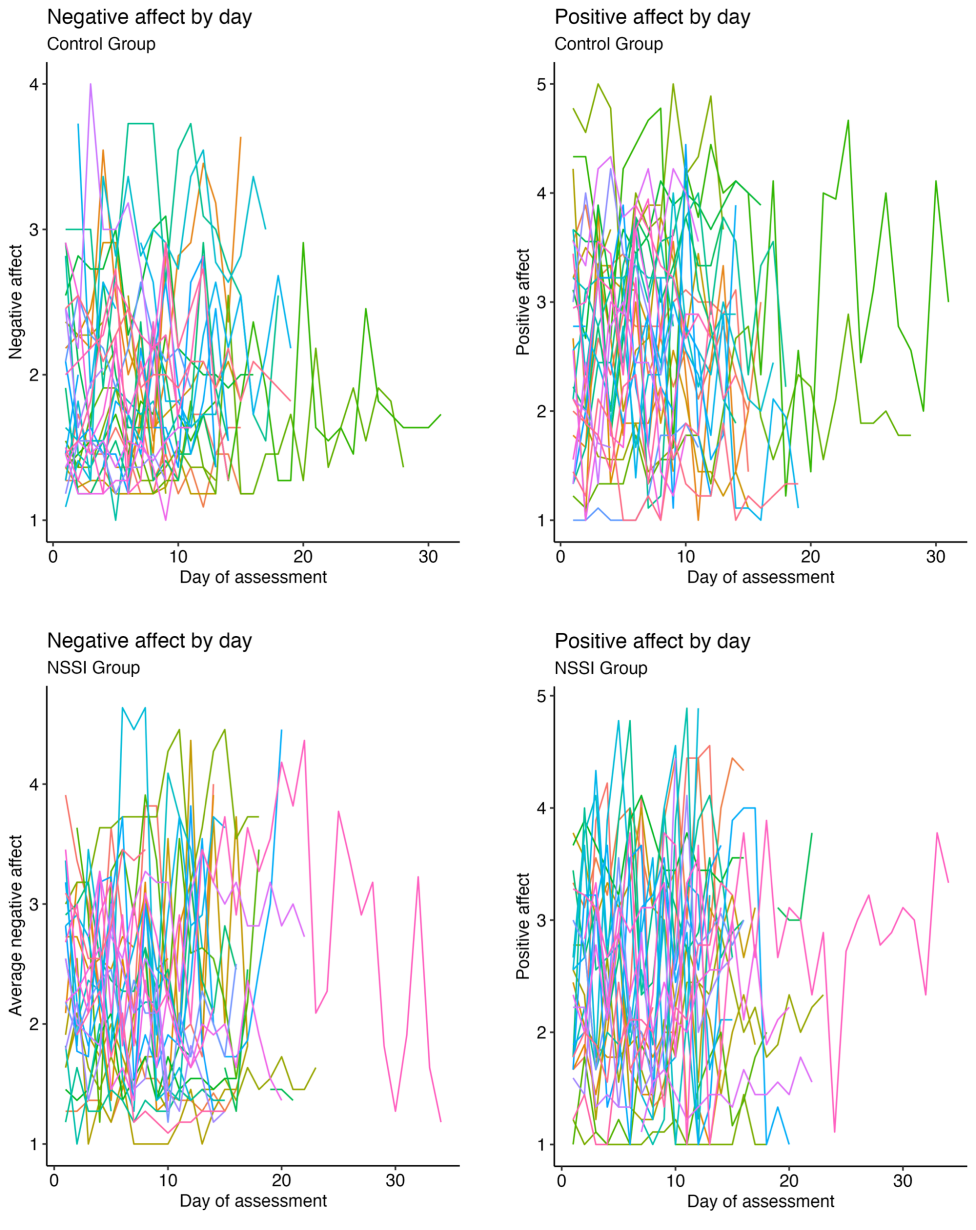
Raw Affect Scores Over the Period of Assessment for All Participants *Note.* Coloured lines represent individual subject’s daily affect assessments; x-axis: number of days of assessment; y-axis: affect intensity.

On average, patients engaged in NSSI showed significantly higher peak complexity scores than controls (Figure 2). This was true for both positive (*t*(44.89) = 2.88, *p* = .006) and negative affect (*t*(57.65) = 2.08, *p* = .042). The effect sizes of these differences were medium for positive affect (*d* = 0.77, 95% CI [0.23, 1.30]) and medium for negative affect (*d* = 0.54, 95% CI [0.01, 1.06]).

 As a robustness analysis, we repeated the comparisons after excluding four patients reporting only between two and five NSSI acts during their lifetime. This led to slightly higher group differences. For positive affect complexity, the difference was significant and large (*t*(42.42) = 3.21, *p* = .002, *d* = 0.86). For negative affect complexity, the difference was significant and medium (*t*(56.26) = 2.17, *p* = .034, *d* = 0.56).

**Figure 3:**
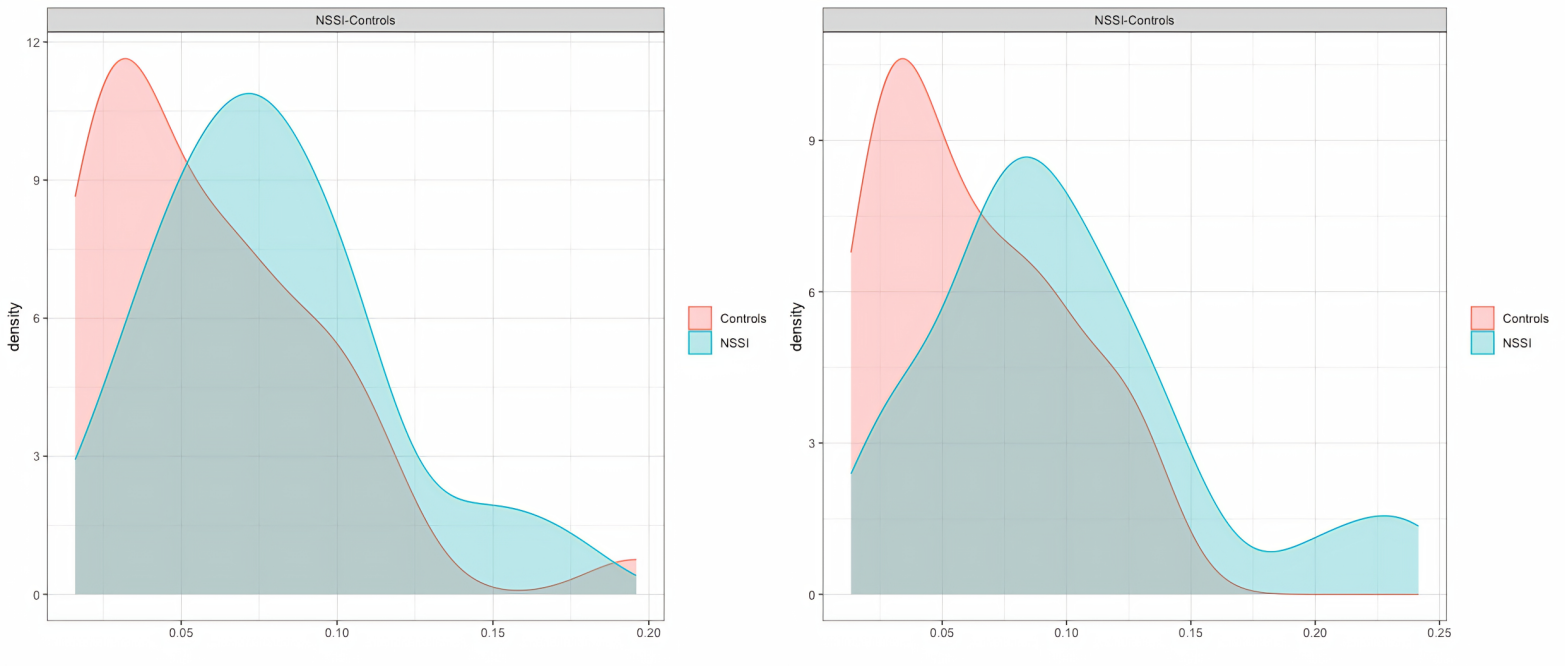
Distribution of Mean Max Complexity *Note.* (A) distribution of mean max complexity of positive affect. (B) distribution of mean max complexity of negative affect.

In addition, NSSI patients had higher mean levels of positive affect: the mean PANAS positive affect score in the NSSI group was 2.41 (*SD* = 0.34) compared to 2.16 (*SD* = 0.36) in the non-NSSI group. This difference was significant (*t*(55,02) = 2,36, *p* = .022; *d* = .61). There was no evidence of differences in negative affect: in the NSSI group, the mean PANAS negative emotion score was 2.26 (*SD* = 0.41), compared to a mean of 2.27 (*SD* = 0.48) in the non-NSSI group (*t*(58.98) = -0.12, *p* = .906). Figure 3 shows the distribution of mean-max complexity in positive and negative affect. The NSSI group has higher scores for both positive and negative affect, demonstrating that the NSSI group generally has higher emotionality than the control group.

Furthermore, we fitted a linear mixed model to predict positive complexity (= DC of positive affect) from time (in days) and group (NSSI vs. control) as predictors. The model included a random intercept for each participant. The model's total explanatory power was substantial (conditional *R*^2^ = 0.50), and the part related to the fixed effects alone (marginal *R*^2^) was 0.02.

The second linear mixed model predicted negative complexity (= DC of negative affect) from the same variables as the first model. The total explanatory power was substantial (conditional *R*^2^ = 0.57), and the part related to the fixed effects alone (marginal *R*^2^) was 0.08. Standardized parameters of the models were obtained by fitting the model on a standardized version of the dataset. 95% Confidence Intervals (CIs) and *p*-values were computed using a Wald *t*-distribution approximation. We did not find an association of length of stay with dynamic complexity. The models are summarized in Table 1 and Table 2.

**Table 1:** Coefficients for the Linear Mixed Model Predicting the Dynamic Complexity of Positive Affect From Time and Group

	Estimates	95% CI	*p*
Predictors			
(Intercept)	0.03	0.02; 0.05	< .001
Days	-0.00	-.00; .00	0.132
Group [NSSI]	0.01	-.01; .03	.333
Days x Group [NSSI]	0.00	-.00; .00	.616
Random Effects			
σ^2^	0.00		
τ_00 Code_	0.00		
ICC	0.49		
*N*_Code_	59		
Observations	671		
Marginal *R*^2^	.02	Conditional *R*^2^	.50

*Note.* 95% CI = 95% confidence interval; NSSI = non-suicidal self-injury; σ^2^ = variance of intercepts; τ_00 Code_ = standard deviation of intercepts; ICC = intraclass correlation; *N*_Code_ = number of participants; Marginal *R*^2^ = variance explained by fixed effects; Conditional *R*^2^ = variance explained by both fixed and random effects.

**Table 2:** Coefficients for the Linear Mixed Model Predicting the Dynamic Complexity of Negative Affect From Time and Group

	Estimates	95% CI	*p*
Predictors			
(Intercept)	0.02	0.01; 0.03	< .001
Days	-0.00	-.00; .00	0.141
Group [NSSI]	0.02	.00; .04	.041
Days x Group [NSSI]	0.00	-.00; .00	.274
Random Effects			
σ^2^	0.00		
τ_00 Code_	0.00		
ICC	0.53		
*N*_Code_	59		
Observations	671		
Marginal *R*^2^	.08	Conditional *R*^2^	.57

*Note.* 95% CI = 95% confidence interval; NSSI = non-suicidal self-injury; σ^2^ = variance of intercepts; τ_00 Code_ = standard deviation of intercepts; ICC = intraclass correlation; *N*_Code_ = number of participants; Marginal *R*^2^ = variance explained by fixed effects; Conditional *R*^2^ = variance explained by both fixed and random effects.

The average reported NSSI frequency was not related to the average positive affect, *r*(26) = -.10, and the average negative affect, *r*(26) = -.04, both *p* > .05. Similarly, there was no significant correlation between the number of NSSIs and the peak complexity for positive (*r*(26) = -.06) and for negative (*r*(26) = .08) emotions, both *p* > .05.

## Discussion

The current study is, to our knowledge, the first to examine DC and the role of positive affect in NSSI, particularly in an adult clinical sample. Consistent with our first hypothesis, participants in the NSSI group showed higher DC in affect – both positive and negative – than controls. This study impressively demonstrated that only a few days are sufficient to show the typical dynamic pattern of high affect fluctuation in individuals with a history of NSSI. Although NSSI is generally associated with an increase in negative affect in most other studies, in our study individuals with a history of NSSI specifically showed more positive affect than controls. We could not find significant differences in mean levels of negative affect. Previous studies have reported mixed findings regarding positive affect in NSSI (e.g., Armey et al., 2011; Jenkins & Schmitz, 2012). We did not find a significant relationship between change in dynamic complexity of affect and length of inpatient stay in either the NSSI or the control group.

The finding that individuals with a history of NSSI showed increased DC for both positive and negative affect is consistent with previous research showing higher affect variability / instability in the NSSI group compared to controls (Santangelo et al., 2017; Victor et al., 2021). The advantage of using DC instead of, for example, classical variance as a measure of varying degrees of fluctuations is that variance is indifferent to the shape of the time series, unlike F and D, which are influenced by it. Variance specifically indicates the magnitude of fluctuations without regard to the frequency or sequence of system states. We were also able to show that the NSSI group generally has higher emotionality than the control group. This result is consistent with other studies based on emotional experience (e.g., Victor & Klonsky, 2014). According to the study by Victor and Klonsky (2014), self-injurers show higher negative emotions than non-injurers.

To explain our finding of increased positive affect in our NSSI group, several explanations can be considered: First, some authors emphasize the addictive nature of NSSI (e.g., Buser & Buser, 2013), including compulsivity, loss of control, and continuation despite negative consequences. In this sense, engagement in NSSI may activate the EOS (endogenous opioid system), which contributes to the experience of analgesia by releasing opiates in response to tissue damage. This physiological process can lead to an improvement in mood (Sandman & Touchette, 2002). Second, positive reinforcement is an important general motive for maintaining NSSI, as demonstrated in previous studies (e.g., Selby et al., 2014). Selby et al. (2014) found that more than 50% of their sample reported at least one instance of NSSI for automatic positive reinforcement (APR) reasons, with "satisfaction" as the most frequently endorsed motivation. In addition, self-injurers with APR motives reported more frequent NSSI. Third, we used a psychiatric control group with a higher proportion of depressed patients (54.55% vs. 42.9%). Depressives typically show elevated levels of negative affect compared to non-depressives. Although we did not test this assumption statistically, it is plausible to assume that the higher proportion of depressives in the control group accounts for the lower levels of positive affect in this group. Fourth, 13 (46.43%) participants in the NSSI group met criteria for a borderline personality disorder diagnosis (primary or comorbid). Borderline personality disorder and NSSI overlap in some criteria (e.g. impaired emotion regulation). Therefore, it could also be possible that the group differences are not due to NSSI, but to other psychopathological characteristics. Future studies should definitely investigate this possibility. Furthermore, Stapleton and Wright (2019) reported that psychiatric inpatients diagnosed with borderline personality disorder indicate positive experiences with inpatient care for many reasons: the opportunity to talk to someone and be listened to, time away from everyday life, feeling safe, and gaining control over their recovery. In our opinion, this argument is particularly important for our study, because almost 50% of the NSSI group consisted of patients with borderline personality disorder, while not a single patient in the control group had this diagnosis. It can therefore be assumed that the patients with borderline personality disorder in our study had similar positive experiences during their inpatient stay. The increased level of positive affect found in our NSSI sample could therefore be attributed to this. Finally, we examined whether average dynamic complexity changed over time. The association was not significant. The average length of stay in the psychiatric department of the Kardinal Schwarzenberg Clinic is between 2 and 3 weeks, and the average assessment period was 17 days for the NSSI group and 14 days for the control group. Perhaps the length of stay or assessment period was too short to detect a change in dynamic complexity over time. Fowler et al. (2016) found significant improvements in experiential avoidance and emotion dysregulation only after 6-8 weeks of intensive inpatient psychiatric treatment.

### Limitations

Our study has several limitations. The first is that our sample size is not very large, which limits generalizability. On the other hand, the results suggest that intra-individual differences in dynamic complexity explain significantly more variance than differences in assessment duration or between-group differences. Future studies with larger samples and, thus, higher power to detect smaller effects, should be done to replicate our findings. A second major limitation is that we made only one measurement per day. Normally, affective changes occur several times per day, especially in emotionally unstable subjects. Consequently, a single assessment per day may not have been sufficient to capture the dynamics of emotional change. Typical ambulatory assessment studies therefore perform several measurements per day. However, this limitation may also be an advantage, as studies have found lower compliance rates in these designs. In their systematic review, Hepp et al. (2020) found an average compliance rate of 64.9% with prompts for more than one daily measurement, while a single daily measurement resulted in an average compliance rate of 78.9%. To minimize the risk of low response rates and early study termination, we chose to use a single measurement per day design. A third possible limitation is that participation in our study was not incentivized. Hepp et al. (2020), in their systematic review of daily life studies of NSSI, found that incentivized studies resulted in higher compliance rates. A fourth limitation is the assessment of the frequency of NSSI. We could only ask our participants for a retrospective estimate of the number of times they had injured themselves in their lifetime. This type of assessment is problematic because individuals may have had difficulties in recalling the exact number of lifetime self-injurious acts. In addition, many members of the NSSI sample have engaged in NSSI continuously over a long period of their lives. In this case, the exact number of NSSI events is likely to be impossible to assess. Due to the lack of compliance, it was not possible to assess individual acts of NSSI during the clinic stay or the survey period. Thus, we could not directly examine associations between acts or attempts of NSSI and affective states. As a result, we could not examine the relationship between emotions and NSSI acts, e.g., whether positive (or negative) affect occurs before or, in the case of a positive act, after an NSSI act. We were unable to assess the effects of the medications that participants received during their stay, as well as the effects of their psychological treatments on their affect fluctuations.

### Conclusion and Implications

The results of our study illustrate that individuals with a history of NSSI show greater dynamic complexity for positive as well as negative affect and higher mean levels of positive affect. Future studies should explicitly consider this possibility and explore the dynamics of positive emotions in the context of NSSI more intensively, especially with larger samples, but also in individual case studies as a second very insightful strategy. The higher variance explained at the individual level suggests that the application of dynamic complexity is more meaningful when considered at the individual case level. Regarding prevention and treatment, future research should also focus on specific patterns of affect dynamics to identify early warning signs of critical instability and order transitions in individuals with a history of NSSI. Knowledge of these phenomena offers the possibility of early intervention in affective change. For example, van de Leemput and colleagues (2014) reported that specific dynamic patterns of emotions are associated with the onset and offset of episodes of major depression. In addition, anticipating and observing order transitions in affect may be useful for clinical assessment of mental disorders and treatment planning (e.g., dialectical behavior therapy (DBT)) to reduce affective instability).

## Supplementary Materials

The Supplementary Materials contain the preregistration for the study (for access, see Bruckbauer-Schwed et al., 2023S).



Bruckbauer-SchwedM.
KaiserT.
PlöderlM.
LaireiterA.-R.
 (2023S). Dynamic complexity of positive and negative affects in NSSI – A daily diary study
[Preregistration]. PsychOpen. 10.23668/psycharchives.13937
PMC1215222740510543

## Data Availability

The data that support the findings of this study are available on request from the corresponding author, Michaela Bruckbauer-Schwed.

## Reporting Guidelines

JARS-Quant guidelines for quantitative research
